# Cost comparison of MRSA screening and management – a decision tree analysis

**DOI:** 10.1186/1472-6963-12-438

**Published:** 2012-12-01

**Authors:** Andrea Tübbicke, Claudia Hübner, Nils-Olaf Hübner, Christian Wegner, Axel Kramer, Steffen Fleßa

**Affiliations:** 1 Institute of Health Care Management, University of Greifswald, Friedrich-Loeffler-Str. 70, 17489, Greifswald, Germany; 2 Institute of Hygiene and Environmental Health, University Hospital, Greifswald, Germany; 3 Division 14: Applied Infection Control and Hospital Hygiene, Robert Koch-Institute, Berlin, Germany

## Abstract

**Background:**

Methicillin-resistant *Staphylococcus aureus* (MRSA) infections represent a serious challenge for health-care institutions. Rapid and precise identification of MRSA carriers can help to reduce both nosocomial transmissions and unnecessary isolations and associated costs. The practical details of MRSA screenings (who, how, when and where to screen) remain a controversial issue.

**Methods:**

Aim of this study was to determine which MRSA screening and management strategy causes the lowest expected cost for a hospital. For this cost analysis a decision analytic cost model was developed, primary based on data from peer-reviewed literature. Single and multiplex sensitivity analyses of the parameters “costs per MRSA case per day”, “costs for pre-emptive isolation per day”, “MRSA rate of transmission not in isolation per day” and “MRSA prevalence” were conducted.

**Results:**

The omission of MRSA screening was identified as the alternative with the highest risk for the hospital. Universal MRSA screening strategies are by far more cost-intensive than targeted screening approaches. Culture confirmation of positive PCR results in combination with pre-emptive isolation generates the lowest costs for a hospital. This strategy minimizes the chance of false-positive results as well as the possibility of MRSA cross transmissions and therefore contains the costs for the hospital. These results were confirmed by multiplex and single sensitivity analyses. Single sensitivity analyses have shown that the parameters “MRSA prevalence” and the “rate of MRSA of transmission per day of non-isolated patients” exert the greatest influence on the choice of the favorite screening strategy.

**Conclusions:**

It was shown that universal MRSA screening strategies are far more cost-intensive than the targeted screening approaches. In addition, it was demonstrated that all targeted screening strategies produce lower costs than not performing a screening at all.

## Background

Methicillin-resistant *Staphylococcus aureus* (MRSA) infections represent a serious challenge for health care institutions [1-3]. In order to assure rapid treatment of MRSA patients, a reduction of unnecessary isolation precautions and prevention of potential cross infections as well as rapid and precise identification of MRSA are required. Although the implementation of MRSA screening is associated with high expenses for the hospital, the cost-effectiveness of performing MRSA screening has already been confirmed in several studies [4-7]. However, MRSA screenings remains a controversial issue, as the identification of the strategy which causes the lowest expected cost for a hospital is still the object of inquiry.

### Who

Which patient groups should be included in the screening program? A mandatory universal screening program includes all inpatients being admitted to the hospital. Targeted screening is limited to high-risk patients or areas of the hospital. Among these high-risk admissions are old and multi morbid patients, patients with chronic diseases, open wounds, eczema, burns, and patients requiring dialysis. Further documented risk factors are long hospital stays, intravenous drug use, invasive lines or tubes such as catheters, and prior antibiotic exposure [8,9]. The Robert Koch-Institute defines the following patient groups as high-risk patients: patients with nursing care dependency, dialysis patients, invasive lines or tubes such as catheters, patients with chronic wounds, burns, skin ulcers, gangrene soft tissue infection as well as patients with known MRSA anamnesis [8,9]. In addition to the criteria named above, patients transferred from regions, hospitals and other medical institutions with a noted high MRSA prevalence, patients with a stay at another hospital during the last three months as well as patients working in animal breeding belong to the high-risk patient group. Furthermore certain areas of the hospital are declared as high-risk areas such as ICU, weaning ward, stroke unit and dermatological ward.

### How

Which laboratory technique allows a rapid, precise and cost-effective diagnostic investigation? After swabs are taken from the patient, hospitals can settle for the polymerase chain reaction (PCR) method, which is a genotypic method to determine the methicillin-resistance of staphylococcus. Another option is the conventional culturing technique. Microorganisms are incubated on culture media and are differentiated phenotypically. Susceptibility to antimicrobials is determined afterwards by standardized methods. Hospitals can also decide for a combination of the two methods.

Another question that needs to be answered is how to deal with screened patients until the MRSA test result is available, that is, should screened patients be treated as MRSA carriers and put in isolation until MRSA is excluded or should no action be taken until MRSA has been confirmed?

### Where

Which swab site or rather a combination of swab sites offers high sensitivity? Nasal swabs are considered to be the minimum standard. Groin, axilla or wound swabs are also appropriate [10].

### When

Is it reasonable to screen patients only upon hospital admission or to additionally conduct follow-ups and contact-patient screenings?

The aim of this study was a cost analysis to determine which MRSA screening and management strategy causes the lowest expected cost for a hospital. For this purpose, a decision tree was modelled and corresponding calculations were made. We conducted various sensitivity analyses in order to examine the stability of the results obtained.

## Methods

### Decision making problem and decision tree

**Figure 1:**
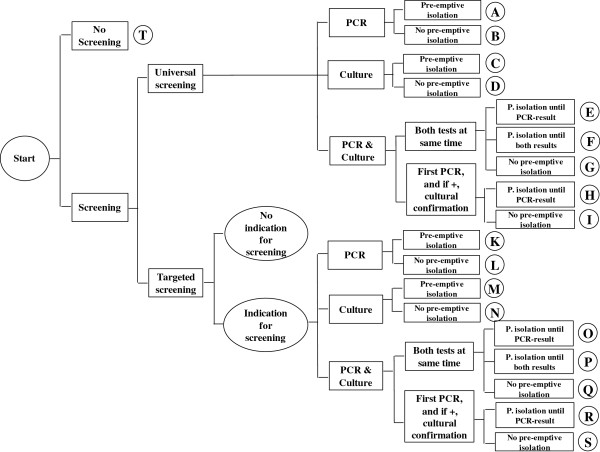
Decision tree.

The decision-making process regarding the selection of a suitable screening strategy has a complex structure. A number of sub-decisions lead to different alternatives. The first branch in the decision tree results from the basic options to screen or not to screen. Following the branch pro-screening, it has to be clarified which patient groups should be included in the screening program. Should a universal screening of all inpatients be conducted or a targeted screening program limited to high-risk admissions or areas of the hospital? Hospitals can choose the PCR methods or culture techniques, or a combination of the two methods. If the institution has chosen the last option, it must be determined next whether both tests should be conducted at the same time or if the culture test should only be applied after a PCR test has yielded a positive result, in order to exclude a false-positive result. The last question that must be answered is how to deal with screened patients until the MRSA test result is available (pre-emptive isolation or not). Out of these different options in the decision-making process, 19 alternative strategies arise. Each strategy is represented by an uppercase letter, A-T (Figure 1).

### Assumptions

**Figure 2:**
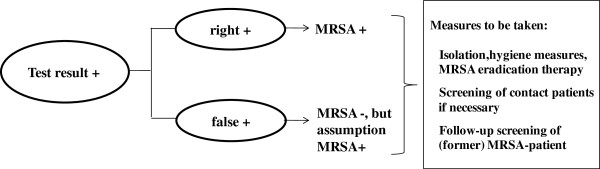
Measures to be taken in case of an MRSA-positive test result.

A positive screening result is always interpreted as true MRSA, although at first it remains unknown whether the test is true-positive or false-negative. For this reason, isolation precautions, hygiene, and MRSA eradication measures must be initiated immediately, which is in keeping with the recommendations of the Robert Koch-Institute [11]. In the present analysis, taking these steps in the event of a positive test result is therefore not regarded as an elective action but as obligatory (Figure 2). Furthermore, it is assumed that screening contact patients is only necessary if no pre-emptive isolation of the MRSA patient was conducted until the test result was available.

If the MRSA-test is negative, pre-emptive isolation is immediately discontinued and no further steps are taken. In the present model, the decision tree branches end after MRSA is excluded or after MRSA eradication treatment and follow-up screening has been conducted. It is assumed that the culture method is applied for the follow-up screening as well as the contact-patient screening and that these test results turn out to be negative. Therefore, costs for a negative culture test are assessed in the calculations. With regard to MRSA prevalence, an interrelation between the parameters “MRSA prevalence inpatients” and “MRSA prevalence high-risk inpatients” is implied. The higher the prevalence among all inpatients, the higher will also be the prevalence among high-risk inpatients and vice versa. However, the exact ratio between the two parameters is unknown.

### Systematic literature search

Several variables (Table 1) and constants (Table 2) are considered relevant for the decision-tree analysis as they have decisive significance for the burden of MRSA and the selection of a specific MRSA screening strategy. In order to obtain concrete values and significances of these factors a systematic literature search for peer-reviewed publications was conducted. The electronic databases PubMed and Web of Science were searched systematically for studies published in German and English between 1995 and April 2011. Searches were also done manually with reference lists from these papers. The process of data acquisition is described in much more detail in a paper that has been published recently [12].

**Table 1:** Definition and quantification of variables

**Variable**	**Description**	**Average value**	**Min value**	**Max value**	**References**
*P*_*t*_	MRSA prevalence of all inpatients	3.08%	1.20%	5.30%	[12]
*P*_*r*_	MRSA prevalence of high-risk inpatients	11.94%	3.85%	20.6%	[12]
*P*_*n.i.*_	MRSA prevalence of patients without indication for a targeted screening	0.03%	0.03%	0.03%	[13]
*RT*_*no iso*_	Rate of MRSA transmission not in isolation per day	0.029	0.0014	0.1400	[12]
*RT*_*iso*_	Rate of MRSA transmission in isolation per day	0.003	0.0008	0.0090	[12]
*Sen*_*PCR*_	Sensitivity of PCR method	91.09%	62.50%	100%	[12]
*Sen*_*cul*_	Sensitivity of culture method	88.73%	53.00%	100%	[12]
*SP*_*PCR*_	Specificity of PCR method	95.79%	83.70%	100%	[12]
*SP*_*cul*_	Specificity of culture method	93.23%	68.00%	100%	[12]
${\overset{―}{C}}_{p\;\text{iso}}$	Average costs for pre-emptive isolation per day	62.77 €	9.45 €	145.11 €	[12]
${\overset{―}{C}}_{\text{MRSA}}$	Average costs per MRSA case per day	506.92 €	213.51 €	1,411.44 €	[12]
∅ *LOS*_*MRSA*_	Average length of stay of MRSA patients in days	24.88	18	39	[12]

**Table 2:** Definition and quantification of constants

**Constant**	**Description**	**Value**	**References**
*T*_*PCR*_	turn-around time of PCR method in days	0.29	in-house data
*T*_*cul*_	turn-around time of culture method in days	2.5	in-house data
*C*_*PCR*_	costs for a single PCR test	20.50 €	[14]
*C*_*cul pos*_	costs for a single culture test with positive result	24.10 €	[14]
*C*_*cul neg*_	costs for a single culture test with negative result	6.40 €	[14]
*C*_*f*_	costs for follow-up screening	6.40 €	As follow-up screening in the majority of cases leads to a negative result, costs for negative culture tests are assessed
*C*_*c*_	costs for screening a contact patient	6.40 €	As follow-up screening in the majority of cases leads to a negative result, costs for negative culture tests are assessed
*Pat*_*c*_	number of contact patients	2	Calculation based on in-house data
∅ *LOS*_*reg*_	average length of stay of regular patients in days	8	Federal Statistical Office [15]
*Pat*_*t*_	number of total inpatients per year	35,322	in-house data
*Pat*_*r*_	number of high-risk patients per year	4,367	in-house data
*Pat*_*n.i.*_	number of patients without indication for a targeted screening per year	30,955	in-house data
*C*_*MRSA ici*_	costs for MRSA caused by incomplete MRSA carrier identification		

The literature search resulted in 98 studies, which met all inclusion and exclusion criteria. Of the 98 eligible studies the following number of articles was retrieved for each parameter:

performance of PCR methods: 36

performance of culture methods: 30

MRSA transmission rates: 7

MRSA prevalence in German hospitals: 6

MRSA prevalence of high-risk inpatients or patients in high-risk areas in German hospitals: 3

costs per MRSA case: 11

costs for pre-emptive isolation per day: 5

As 9 of all 98 identified studies provide information for more than one parameter of interest, these studies were retrieved several times. Eventually, 87 different studies were identified by the search. Relevant data were abstracted from all these studies. Tables 1 and 2 provide an overview (minimum, maximum and average values of the retrieved data) of the concrete values and significances of the variables and constants obtained by the literature review.

### Parameters

Since not all parameters compiled in the Tables 1 and 2 could be quantified by systematic literature, assumptions had to be made. The costs for a PCR test as well as for positive and negative culture tests were estimated corresponding to the German uniform valuation standard (EBM) [14]. Estimations of the turn-around times of both methods are based on in-house data. The number of contact patients was calculated by dividing the average occupied hospital beds by the number of patient rooms.

### Calculation

The decision model calculates the expected costs for each scenario, E(X). This principle is demonstrated by the formula for the strategy D.

$$\begin{array}{l} E\left(D\right)=\\ Pat_{t\;}\cdot P_{t\;}\cdot Sen_{\text{cul}}\\ \;\cdot C_{\text{cul}\;\text{pos}}+T_{\text{cul}}\cdot RT_{\text{no}\;\text{iso}}\cdot {\overset{―}{C}}_{\mathrm{MRSA}}\cdot \emptyset LOS_{\mathrm{MRSA}}\\ \;+C_{c}\cdot Pat_{c}++\left(\emptyset LOS_{\mathrm{MRSA}}-T_{\mathrm{cul}}\right)\\ \;\cdot RT_{\mathrm{iso}}\cdot {\overset{―}{C}}_{\mathrm{MRSA}}\cdot \emptyset LOS_{\mathrm{MRSA}}\\ \;+{\overset{―}{C}}_{\mathrm{MRSA}}\cdot \emptyset LOS_{\mathrm{MRSA}}+\\ \left[Pat_{t}\cdot \left(1-P_{t}\right)\cdot \left(1-SP_{\mathrm{cul}}\right)\right]\cdot C_{\text{cul}\;\text{pos}}+C_{c}\cdot Pat_{c}+C_{f}+\\ \;{\overset{―}{C}}_{\mathrm{MRSA}}\cdot \emptyset LOS_{\mathrm{reg}}+\\ \left[Pat_{t}\cdot \left(1-P_{t}\right)\cdot SP_{\mathrm{cul}}\right]\cdot C_{\text{cul}\;\text{neg}}+\\ \left[Pat_{t}\cdot P_{t}\;\cdot \left(1-Sen_{\mathrm{cul}}\right)\right]\cdot C_{\text{cul}\;\text{neg}}+\\ \;\emptyset LOS_{\mathrm{MRSA}}\cdot RT_{\text{no}\;\text{iso}}\cdot {\overset{―}{C}}_{\mathrm{MRSA}}\cdot \emptyset LOS_{\mathrm{MRSA}} \end{array}$$

Congruent to the procedure shown above, the expected costs for each Strategy A- T are determined.

### Sensitivity analyses

A multiplex sensitivity analysis was conducted by a simultaneous variation of more than one parameter. The following variables were varied in three steps from minimum to maximum of the existing data (Table 1): rate of MRSA transmission not in isolation per day, rate of MRSA transmission in isolation per day, costs for MRSA case per day, costs for pre-emptive isolation per day, MRSA prevalence inpatients, MRSA prevalence high-risk inpatients, sensitivity of PCR method, specificity of PCR method, sensitivity of culture method and specificity of culture method. Consequentially, 59,049 (= 3^10^) different scenarios were generated.

Single sensitivity analyses aimed at determining the influence of a certain parameter on the expected costs of the screening strategies. The parameter in focus was therefore increased in ten steps from minimum to maximum of the existing data according to Table 1 while all other variables were kept at their average value.

## Results

### Basic analysis

**Figure 3:**
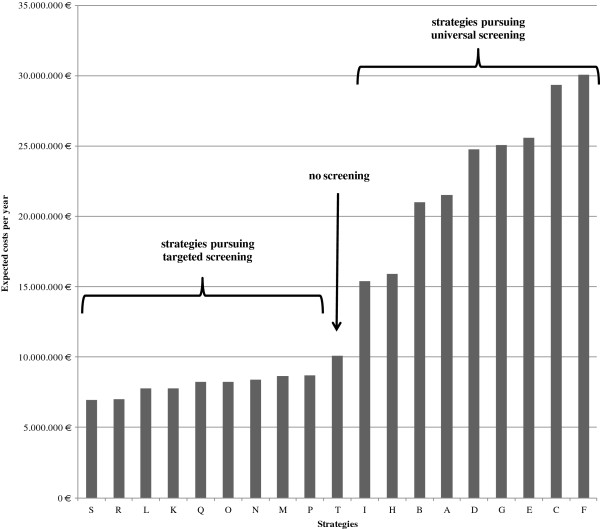
Basic analysis: Expected costs for strategies.

As presented in Figure 3 targeted screening strategies show the lowest expected costs. Among these strategies, a combination of PCR and culture method or performing the MRSA test via PCR is advantageous in comparison to the application of the culture method alone. Strategy S, representing a targeted PCR screening and culture confirmation in the case of a positive PCR result and no pre-emptive isolation causes the lowest expected costs. This approach is closely followed by Strategy R, which also uses a targeted PCR screening with culture confirmation in the case of a positive PCR result, but carries out pre-emptive isolation precautions. Among all targeted strategies, the most expensive approach is the implementation of PCR and the culture method at the same time and performing pre-emptive isolation of screened patients until both test results are available.

The results show that an omission of an MRSA screening causes higher costs than performing a targeted screening. Only a universal screening approach is more costly than not to perform a screening at all.

### Multiplex sensitivity analysis

**Figure 4:**
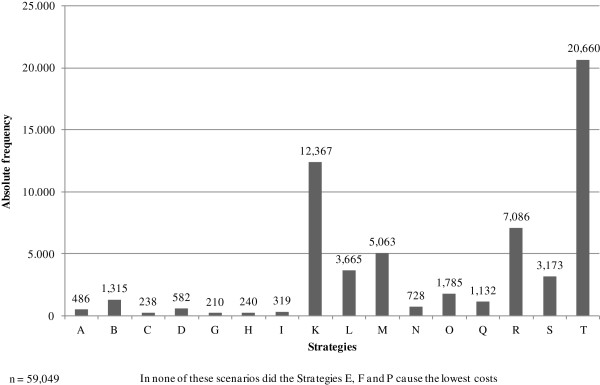
Multiple sensitivity analysis: Absolute frequency of causing the lowest costs.

An analysis was performed to determine in how many of the 59,049 scenarios the respective strategies produced the lowest costs. The results are presented in Figure 4. In 20,660 cases, Strategy T (no screening) causes the lowest costs. In 12,367 scenarios, Strategy K (targeted screening by PCR method and pre-emptive isolation) leads to the lowest expected costs. In 7,086 out of 59,049 cases Strategy R (targeted PCR screening with culture confirmation in the case of a positive PCR result and pre-emptive isolation) is the best alternative. In agreement with the results of the basic analysis, targeted screening strategies are more cost-saving than the universal screening strategies, since they more often caused the lowest costs.

**Figure 5:**
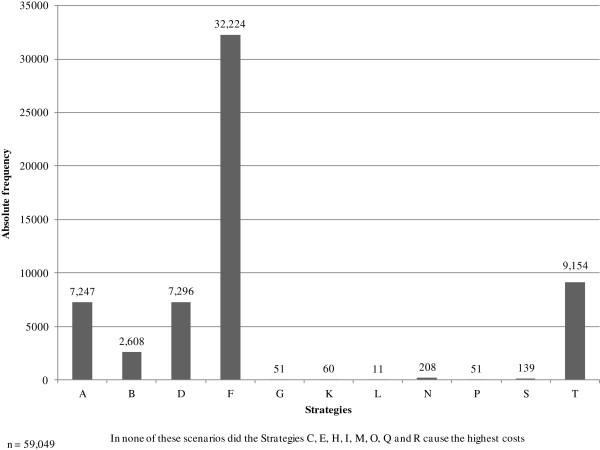
Multiple sensitivity analysis: Frequency of causing the highest costs.

In addition, an analysis was performed to determine in how many cases the respective strategies cause the highest expected costs (Figure 5). With an absolute frequency of 32,224 scenarios, Strategy F (universal screening by simultaneously conducting PCR and the culture method and performing pre-emptive isolation precautions until both results are available) most frequently shows the highest expected costs compared to all other alternatives, which corresponds to the results of the basic analysis. Not to perform an MRSA screening (Strategy T) is the second-worst choice the decision-maker can take, followed by the universal strategies D, A and B. All other alternatives show a low frequency of causing the highest expected costs.

### Single sensitivity analysis

As expected, the increase of the parameter “costs per MRSA case per day” led to growing costs. Over the entire augmentation of this parameter strategies R and S (targeted PCR screening with culture confirmation in the case of a positive PCR result with and without pre-emptive isolation) proved to be the cost-minimizing screening strategies. These strategies showed almost equal outcomes. However, Strategy S was favourable at amounts up to 740 €, while Strategy R became advantageous when costs per MRSA case per day were higher.

The increase of the parameter “costs for pre-emptive isolation per day” resulted in an increase of costs for all strategies which include the conduction of pre-emptive isolation precautions, while the costs for all other alternatives remained constant. The Strategies R and S once again produced the lowest costs. Up to costs amounting to 44 €, Strategy R was the better choice. If the costs for pre-emptive isolation per day were higher than that, the decision-maker should choose Strategy S.

**Figure 6:**
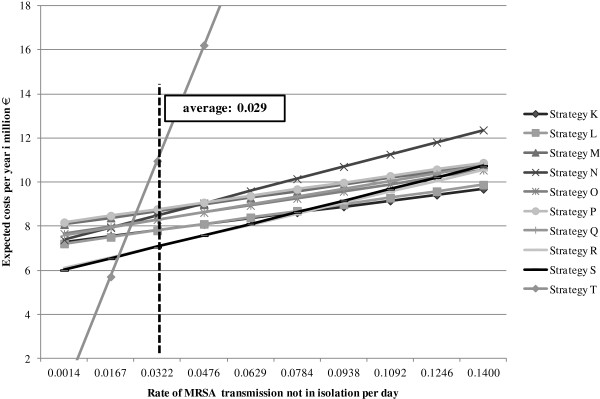
Single sensitivity analysis: Rate of MRSA transmission not in isolation per day.

A continuous increase of the parameter “MRSA rate of transmission not in isolation per day” also resulted in rising costs for all examined strategies. It was evident that the costs of the alternatives which pursue pre-emptive isolation precautions showed a minor increase compared the other strategies (Figure 6). The greatest increase in costs is observed for Strategy T (no screening). Although cost-minimizing at a low rate of MRSA transmission, this approach became more and more expensive in the course of parameter increase. Up to a transmission rate of 0.0154, not performing a screening produced the lowest costs. From this rate on to 0.042, Strategy S (targeted PCR screening with culture confirmation in the case of a positive PCR result and no pre-emptive isolation) proved to be the best choice. If the rate of transmission ranged from 0.042 to 0.0815, performing a targeted PCR screening and a culture confirmation if needed as well as pre-emptive isolation precautions (Strategy R) was the most favourable option. If the transmission rate was higher than that, Strategy K (PCR screening with pre-emptive isolation) caused the lowest costs for the hospital.

**Figure 7:**
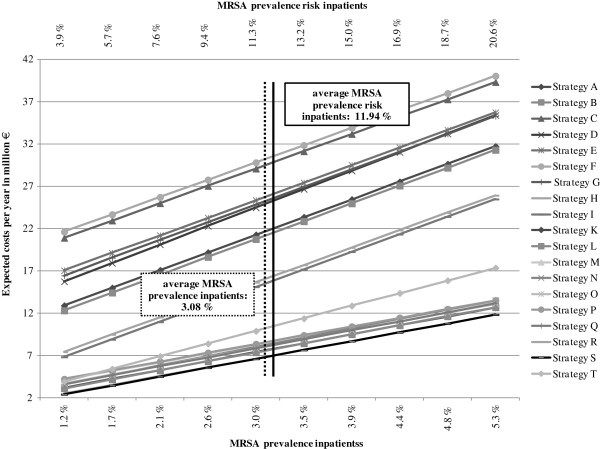
Single sensitivity analysis: MRSA prevalence inpatients and MRSA prevalence high-risk inpatients.

The parameters “MRSA prevalence inpatients” and “MRSA prevalence high-risk inpatients” were increased separately in ten steps. Taking into account the interrelation of these parameters, the effects are illustrated in only one diagram (Figure 7). Increasing MRSA prevalence (all inpatients and high-risk inpatients) leads to higher costs for all strategies. In comparison to the targeted screening approaches, the universal screening strategies produce higher costs without exception. At a low MRSA prevalence, Strategy T (no screening) produces costs comparable to the targeted screening strategies. Costs for this alternative become disproportionately high when MRSA prevalence increases. Once again, Strategies S and R prove to be the cost-minimizing ones. Up to an MRSA prevalence of 16.9% among high-risk inpatients, Strategy S (targeted PCR screening with culture confirmation in the case of a positive PCR result and no pre-emptive isolation) is the cheaper alternative. If the MRSA prevalence among high-risk inpatients is higher than that, choosing the same approach but performing pre-emptive isolation is recommended (Strategy R).

## Discussion

To the authors’ knowledge, this study is the first quantitative evaluation to determine which MRSA screening and management strategy causes the lowest expected cost for a hospital based on a decision-tree model. We succeeded in identifying who, how, when and where to screen patients for MRSA in order to minimize costs.

The results of the decision-tree analysis suggest that a universal screening approach cannot be expected to be cost-minimizing. All screening strategies pursuing a screening for all inpatients produce higher costs than not to perform a screening at all. This is congruent with the results of other studies, which state that screening all patients being admitted to the hospital is desirable in terms of a complete carrier identification, but this approach is neither affordable nor cost-reducing for hospitals [16,17].

Furthermore, it was shown that all strategies performing a targeted screening produce lower costs in comparison to an omission of MRSA screening. For this reason, evidence for the advantageousness of conducting a targeted screening for all high-risk patients is provided by cost-analyses. This is consistent with results of other analyses [4-7,18].

Choosing Strategy T, which means not performing a screening, implies the highest risk for the hospital. Under certain circumstances it can be advantageous, but oftentimes it is the most expensive alternative and on average considerably worse than pursuing a targeted screening approach. Risk-averse decision-makers would therefore tend not to choose this option.

The results show that the alternatives S and R produce the lowest expected costs among all screening strategies. Both strategies focus on PCR screening with culture confirmation in the case of a positive PCR result. The difference between the two options refers to pre-emptive isolation, which is performed by Strategy R and omitted by Strategy S. While both alternatives on average cause costs in similar amounts, the decision-maker should select Strategy R, because it is often the cheapest but never the most expensive. The advantage can be ascribed to the pre-emptive isolation precautions, which minimizes the risk of MRSA cross transmissions and at the same time contains the costs for the hospital. This increases in relevance when the rate of MRSA transmission not in isolation and MRSA prevalence turn out to be high. This approach is also supported by the literature. When a PCR result proves to be positive, the possibility remains that it is false-positive, because the validity of the PCR method is restricted in the case of a coincidental colonization with *Staphylococcus aureus* and coagulase-negative staphylococcus [16]. Furthermore, the PCR method can lead to positive test results even when MRSA germs have been inactivated by a successful eradication therapy [19]. For this reason, it is recommended to perform a culture confirmation, in order to exclude false-positive PCR results [20].

Our analysis has various limitations. First, a weakness can be seen in the quantification of the parameters included in the calculations. Our data were basically obtained by a systematic literature research [12], but in some cases, the data were insufficient and assumptions had to be made on the basis of in-house data and experience. The better the data are tailored to the particular hospital setting, the more significant are the results produced by the decision-tree analysis modelled for this study. In this context, a concrete limitation is evident in terms of the single sensitivity analysis of MRSA prevalence. It is known that there is an interdependence of the parameters “MRSA prevalence”, “MRSA prevalence high-risk inpatients” and “MRSA prevalence among patients without indication for a targeted screening”, but the exact ratio remains uncertain, which limits the validity of this single sensitivity analysis [13].

The results of our decision-tree analysis and the corresponding calculations still allow us to draw conclusions and give recommendations on how to perform an MRSA screening in the cost-minimizing way.

## Conclusions

In summary, it was shown that universal MRSA screening strategies are far more cost-intensive than the targeted screening approaches. In addition, it was demonstrated that all targeted screening strategies produce lower costs than not performing a screening at all. The advantageousness of performing a targeted MRSA screening is therefore proven by the present analysis. The omission of an MRSA screening was identified as the alternative attended by the highest risk for a hospital. Among the targeted screening strategies, performing a PCR screening and culture confirmation in the case of a positive PCR result (strategies R and S) generates the lowest costs for a hospital. The decision-maker should select Strategy R, as pre-emptive isolation minimizes the chance of false-positive results as well as the possibility of MRSA cross transmissions, and at the same time contains the costs for the hospital. Therefore, it bears a lower risk in comparison to Strategy S, which does not take pre-emptive isolation precautions.

Further evidence, especially empiric data on costs and outcomes, would help to strengthen the model and support the hitherto largely theoretical statements.

## Competing interests

The authors declare that they have no competing interests.

## Authors’ contributions

AT, CH and NOH conceptualized the study. AK and SF were involved in the study design. AT and CH collected data by literature researches. CW and AT performed the mathematical analysis. AT and CH drafted the manuscript. All authors read and approved the final manuscript.

## Pre-publication history

The pre-publication history for this paper can be accessed here:

http://www.biomedcentral.com/1472-6963/12/438/prepub
